# Inhibition on JNK Mimics Silencing of Wnt-11 Mediated Cellular Response in Androgen-Independent Prostate Cancer Cells

**DOI:** 10.3390/biology9070142

**Published:** 2020-06-27

**Authors:** Elif Damla Arisan, Ozge Rencuzogullari, Buse Keskin, Guy H. Grant, Pinar Uysal-Onganer

**Affiliations:** 1 Gebze Technical University, Institute of Biotechnology, 41400 Gebze-Kocaeli, Turkey; damlaarisan@gmail.com; 2 Istanbul Kultur University, Department of Molecular Biology and Genetics, Atakoy Campus, 34156 Istanbul, Turkey; ozgeberrak@gmail.com (O.R.); keskinbuse19@gmail.com (B.K.); 3 School of Life Sciences, University of Bedfordshire, Park Square, Luton LU1 3JU, UK; guy.grant@beds.ac.uk; 4 Cancer Research Group, School of Life Sciences, College of Liberal Arts and Sciences, University of Westminster, 115 New Cavendish Street, London W1W 6UW, UK

**Keywords:** c-Jun signalling, prostate cancer, apoptosis, Wnt-11, neuroendocrine-like differentiation

## Abstract

Prostate cancer (PCa) is one of the most common cancers among men, and one of the leading causes of cancer death for men. The c-Jun N-terminal kinase (JNK) pathway is required for several cellular functions, such as survival, proliferation, differentiation, and migration. Wnt-11, a member of the Wnt family, has been identified for its upregulation in PCa; however, downstream signalling of Wnt-11 remains to be fully characterized. In this study, we investigated the role of the JNK pathway as a potential downstream factor for Wnt-11 signalling. For this purpose, LNCaP, DU145, and PC-3 PCa cells and normal epithelial PNT1A cells were treated with a specific JNK kinase inhibitor: JNKVIII. Our results showed that JNK inhibition decreased mitochondrial membrane potential and promoted cell death in a cell type-dependent manner. We found that JNK inhibition led to an increase in autophagy and prevented epithelial–mesenchymal transition (EMT) in independently growing androgen cells. JNK inhibition and the silencing of Wnt-11 showed similar responses in DU145 and PC-3 cells and decreased metastasis-related biomarkers, cell migration, and invasion. Overall, our results suggest that JNK signalling plays a significant role in the pathophysiology of PCa by mediating Wnt-11 induced signals. Our data highlights that both the JNK pathway and Wnt-11 could be a useful therapeutic target for the combinatory application of current PCa.

## 1. Introduction

Prostate cancer (PCa) is the second most common cancer in males [1]. It is a complex disease of an unpredictable nature, with a 32% survival rate [2]. PCa develops mainly in aged men, with an inherited risk of 60% [3], and predisposing genes have been identified [4,5]. Other risk factors include race, a diet rich in fat, and obesity [6]. There are no reliable prognostic markers to identify which tumours are likely to cause aggressive metastatic disease, and this complicates treatment decisions. Therefore, an improved understanding of the genetic and biologic mechanisms determining why some PCa cases remain silent while others become a serious illness is urgently needed. The c-Jun N-terminal kinases (JNKs) orchestrate several signalling pathways as cellular responses to many types of stresses, controlling the proliferation, differentiation, survival, and migration of specific cell types [7]. JNKs belong to the family of mitogen-activated protein kinases (MAPKs). Their short-term activation is required for cell survival, whereas more sustained activation of JNKs causes pro-apoptotic signalling [8]. JNK participates in mRNA stabilization, cell migration, and integrity of the cell skeleton [9], and members of the JNK family are robustly involved in the regulation of various transcriptional factors, such as ATF2, STAT3, c-Myc, and the Bcl-2 family [10,11,12,13]. Three genes were identified in the JNK family—JNK1 (MAPK8), JNK2 (MAPK9), and JNK3 (MAPK10)—and together they encode 10 different splice variants [14]. JNKs are also required for the growth of prostate carcinoma in vitro and in vivo, which makes the JNK pathway a novel target for the treatment of PCa [7].

It is well established that the androgen receptor (AR) plays a crucial role in triggering and progressing PCa, and is currently targeted routinely in clinics to cure PCa. Previous studies have suggested that JNKs could modulate ARs via various pathways, and it was shown that JNK deficiency may cause the development of androgen-independent cancer cells in mice models [15]. In addition, JNK may promote the formation of prostate intracytoplasmic lesions, and may function as an effector of increased PI3K/AKT signalling caused by PTEN inactivation. Besides these findings, JNK inhibitor AS602801 promoted the apoptotic efficiency of enzalutamide by blocking AR expression [16]. However, the cellular responses against JNK inhibitors are not well clarified, especially in androgen-independent cells. The Wnt signalling pathway has been identified to be crucial during tumorigenesis of many malignancies, as well as PCa, and can modulate AR signalling [17]. It has been reported that PCa cells that express non-canonical Wnts may survive longer when AR signalling is blocked [18].

Wnt-11, a non-canonical Wnt signalling family member, is known to play a significant pathophysiological role in major carcinomas, including prostate, cervical, ovarian, and colon [19,20,21]. We have previously demonstrated that the mRNA expression of Wnt-11 was elevated in PCa [20,21,22] both in vivo and in vitro. Furthermore, Wnt-11 has also been associated with neuroendocrine-like differentiation (NED) [23]. Neuroendocrine cells represent a population of cells found within the epithelium of the prostate gland [24] and induce cellular differentiation and growth [25]. Activation of the non-canonical Wnt pathway triggers either the planar cell polarity pathway or the JNK pathway [26]. TGFβ-1 has been identified as a key activator of JNK1, which in turn phosphorylates the cell cycle regulator p21, and up-regulates its stability through a SMAD-independent mechanism [9,27,28]. Wnt-11 is downstream to TGFβ, which has previously been shown to be one of the triggers of the epithelial–mesenchymal transition (EMT), as well as chemoresistance in cancer [29,30]. EMT is a cellular mechanism that is recognized as a normal developmental event; recent evidence suggests that cancer stem cells and EMT-type cells play important roles in chemoresistance and radioresistance [31,32]. Wnt signalling is accepted as one of the regulators of EMT by cross-talking with other signalling pathways [29].

In this study, we aim to analyse further the effect of the JNK pathway on the cellular level by comparing its effect on different PCa cell lines. The results indicate that the inhibition of JNK affects the cell survival and death axis via altering apoptosis, autophagy, and EMT-related signalling targets in cell- and time-dependent manners. Concomitantly, JNK suppression decreased the expression levels of mesenchymal markers and increased epithelial markers; cell behavior assays, such as migration and invasion, suggest that JNK inhibition prevents the metastatic potential of PCa cells in a cell type-dependent manner. Therefore, the JNK pathway is a crucial target to control cell death and the differentiation potential of cancer cells.

## 2. Material and Methods

### 2.1. Cell Culture and Pharmacological Treatments

LNCaP, DU145, PC-3 prostate cancer, and PNT1A prostate epithelial cells were obtained from the American Type Culture Collection (ATCC). The cell lines were maintained in RPMI 1640 medium with 10% foetal bovine serum (FBS) (Invitrogen, Hemel Hempstead, United Kingdom) and 1% penicillin–streptomycin supplemented with L-glutamine. The inhibitor used for the JNK pathway (JNKi) was JNK VIII (Calbiochem, Hertfordshire, United Kingdom), with a working concentration of 10 µM in DMSO.

### 2.2. Cell Cycle and Apoptosis Assays

Cells were seeded in six-well plates at a density of 5 × 10^5^, and were then treated with JNKi for 48 h. Following the incubation, both floating and adherent cells were analysed. Cell cycle analysis was performed on an Accuri C6 (BD Biosciences, Berkshire, United Kingdom). The results obtained from this experiment were from three independent experiments. To assess the apoptosis, we utilized an annexin V–propidium iodide (PI) assay, according to the manufacturer’s instruction (BD Biosciences, Berkshire, United Kingdom). Briefly, cells were resuspended for 10 min in the staining solution, and then analysed by flow cytometry. The percentages of cells were determined from 20,000 cells per sample, by using the FL1 channel for Annexin V and the FL2 channel for PI.

### 2.3. Mitochondrial Membrane Potential Loss by DiOC6 Staining and Mitotracker Red Staining

5 × 10^4^ cells per well were seeded in 12-well plates and treated with JNKi for 48 h. Cells were washed once with PBS and then stained with 0.4 nM DiOC6 before analysing by fluorescence microscopy (Ex/Em: 488/ 525 nm, Olympus IX70). In parallel, cells were stained with Mitotracker Red (Stock solution 1 mM, Thermo Scientific, Hemel Hempstead, United Kingdom), which was diluted 1:1000 and incubated at 37 °C for 45 min. Cells were washed once with PBS and visualized by fluorescence microscopy (Ex/Em: 570/620 nm, Olympus IX70). The experiments were repeated three times.

### 2.4. LysoTracker Red Uptake and Reactive Oxygen Species Detection

50 nM LysoTracker Red (Molecular Probes, Thermo Scientific, Hemel Hempstead, United Kingdom) was applied to the cells for 30 min, and then red lysosomal fluorescence of cells per sample was determined by fluorescence microscopy (Ex/Em: 577/590 nm, Olympus IX70; *n* = 3). To detect reactive oxygen species, cells were seeded onto six-well plates with 1.5 × 10^5^ density, and treated with JNKi for 48 h. H2DCFDA (Thermo Scientific) staining (5 μM) was applied to the cells for 30 min, and then analysed by fluorescence microscopy (Ex/Em: 492/517nm, Olympus IX70; *n* = 3).

### 2.5. Acridine Orange Staining and GFP Transfection

For acridine orange staining, DU145 and PC-3 cells were seeded at 15 × 10^4^ density in coverslips placed in a six-well plate and incubated for 24 h at 37 °C in a CO_2_ incubator. The cells were treated with JNK inhibitor for 48 h. After 48 h, the cells were washed with PBS and stained with 1 µl/mL 3,6-Acridine diamine orange (AO) (5mg/mL stock concentration in DMSO); this was performed for 10 min in the 37 °C incubator. Stained acidic vacuoles (lysosomes, endosomes, and autophagosomes) were determined by fluorescence microscopy in excitation (460 nm) and emission (650 nm).

For GFP–LC3 transfection, DU145 and PC-3 PCa cells were seeded at 1 × 10^5^ density in coverslips placed in a six-well plate. The GFP–LC3 plasmid was transfected into cells with FuGene Transfection Reagent (Promega, Southampton, United Kingdom) in a 3:1 ratio. One µl of plasmid and 3 µl of transfection reagent were placed into serum-free media in two different centrifuge tubes and incubated for 10 min. Then the two tubes were combined and gently pipetted. After pipetting, they were incubated for 20 min. Lysates were added drop wise to the serum media on top of the cells. The cells were incubated for 48 h at 37 °C in a CO_2_ incubator for transfection. GFP–LC3 transfected cells were treated with JNKi (10 µM) at 48 h, and then examined by fluorescence microscopy.

### 2.6. Western Blotting

The total protein samples were extracted and then determined by the Bradford method (Bio-Rad, Hercules, CA, United States), and 12% SDS–PAGE gels were used to identify the protein targets. Following the transfer and blocking, the Polyvinylidene fluoride (PVDF) membranes were incubated with primary antibodies for apoptosis and autophagy, as well as EMT targets: caspases 3 and 7, Atg5, LC3, p62, Beclin-1, E-cadherin, β-catenin, Slug, Vimentin, Dvl-3, Dvl-2, LRP6, and Wnt5a (each 1:1000 from CST (Danvers, MA, USA)), as well as Wnt-11 (1:1000; RnD Systems, Abingdon, United Kingdom). HRP-conjugated, secondary anti-rabbit, anti-mouse, and anti-goat antibodies (CST, 1:3000) were used, and the protein expression was detected using the ChemiDoc Gel Imaging System (Bio-Rad, Hercules, CA, USA).

### 2.7. Real-Time PCR

The total RNA was extracted using the Qiagen mRNA extraction kit, according to the manufacturer’s instructions (Qiagen, Manchester, United Kingdom). RNA quality and quantity were assessed by nanodrop analysis. The cDNA was generated by the reverse transcriptase reaction and used for real-time PCR (qRT-PCR). The following genes were studied: NSE, Asl1, Hes6, Nanog, Twist, Snail, E-cadherin, and Wnt-11 (corresponding primer sequences given previously [32,33]). Analysis by real-time qPCR was performed using Taq SYBR Green premix (Qiagen, Manchester, United Kingdom), as reported before. Relative levels of mRNA expression were calculated using the Comparative CT/2-ΔΔCT method [33,34]. RPII (RNA polymerase II) was used as the reference gene. [34] Experiments were performed three times as biological repeats with triplicate technical repeats, then statistical significance was analysed using a Student’s *t*-test.

### 2.8. siRNA Transfection

PC-3 cells were transfected with siRNA for WNT-11 (Dharmacon, Cambridge, United Kingdom) and non-targeting control (Dharmacon, Cambridge, United Kingdom). The siRNA transfections were performed by using DharmaFECT 2 (Dharmacon, Cambridge, United Kingdom), as described previously [32]. Transfected cells were processed for RNA extraction (after 48 h) or used in cell migration and immunostaining assays (72 h).

### 2.9. Immunostaining

JNKi treated or non-treated control 2 × 10^4^ cells were seeded onto 13 mm sterile coverslips, pre-coated with poly-L-lysine in 24 well plates, and fixed in 4% paraformaldehyde (PFA) for 15 min. Permeabilizing the cells with 0.1% Triton for 15 min at room temperature was followed by a blocking procedure for 30 min. Cells were incubated overnight at 4 °C with the primary antibody, Snail1 (20C8, Thermo Fisher, Hemel Hempstead, United Kingdom), and then with goat anti-mouse IgG (Alexa488, Abcam) secondary antibody diluted 1:500 in the washing buffer. The coverslips were then co-stained with 4’,6-diamidino-2-phenylindole (DAPI) (1.5 g/mL), and wide-field epifluorescence images were acquired at room temperature on an upright fluorescence microscope (Zeiss, Ulm, Germany). The results obtained from this experiment were from three biological repeats.

### 2.10. Hanging Drop Assay

DU 145 and PC-3 PCa cells were counted and calculated to be 2.5 × 10^3^ each drop. The cells were seeded in a 60 mm plate lid as 10 µl drops in the presence or absence of JNK inhibitor (10 µM). Plate lids were gently flipped upside down and placed on a plate filled with 3 mL PBS (to humidify the drops). The cells were incubated at 37 °C in a CO_2_ incubator. Three-dimensional (3D) spheroid formations were examined under a light microscope for 96 h. After 96 h, each drop was stained with DAPI and DIOC6 dyes and examined under fluorescence microscopy.

### 2.11. Migration, Invasion, and Proliferation Assays

Cell migration assays were performed as previously described [20]. Following 6 h incubation, the total number of migrated cells was determined by MTT assay, and this was confirmed by crystal violet assay. In parallel, the same number of cells was plated and incubated for 6 h to determine the effect of cell proliferation by MTT assay. Migration and proliferation were presented as “Migration” and “Proliferation”, i.e., percentage (%) of the readings for invaded cells versus original cell numbers. A Boyden Chamber Assay was used to determine cell migration and invasion through the utilization of Boyden chambers with 8 μm pore track-etched polyethylene terephthalate (PET) membranes (Fisher Scientific, United Kingdom). Cells/well (25 × 10^4^) were seeded after transfection protocols were conducted in a 200 µl serum-free medium in the chamber. Medium (750 µl with 10% serum) was added to the lower chamber. Cells were incubated at 37 °C in CO_2_ for 16 h. Cells were fixed with 3.7% formaldehyde for 2 min at room temperature (RT). Formaldehyde was removed, and cells were washed with PBS. Methanol (100%) was used for permeabilization for 20 min at RT. Cells were washed with PBS twice and stained with crystal violet. Stained cells were visualized by light microscopy at ×10 and ×40 magnification. Cells were counted for five random fields from ×10 magnification and analyzed.

### 2.12. Data Analysis

All data were analysed as means ± standard errors. The student’s *t*-test or ANOVA with Newman–Keuls post hoc analysis was used to determine the statistical significance, as appropriate. Results were considered significant for *p* < 0.05. Western blot results were analysed and normalised to β-actin. Tukey’s multiple comparison tests were used.

## 3. Results

The results are shown in three parts. First, we show that JNKi alters mitochondrial membrane potential and increases cell death in a cell-type dependent manner via reactive oxygen species (ROS) generation. Second, using both siRNA and specific JNKi, we demonstrate that Wnt-11 controls the expression of several biomarkers associated with metastasis. Finally, we show that silencing Wnt-11 expression or blocking the JNK pathway results in a significant decrease in cell migration. Overall, these results suggest that JNK inhibition mimics similar cellular responses following Wnt-11 silencing, which plays a significant role in the pathophysiology of PCa via JNK signalling.

### 3.1. Blocking JNK Pathway Alters Cellular Fate in a Cell Type-Dependent Manner

**Figure 1:**
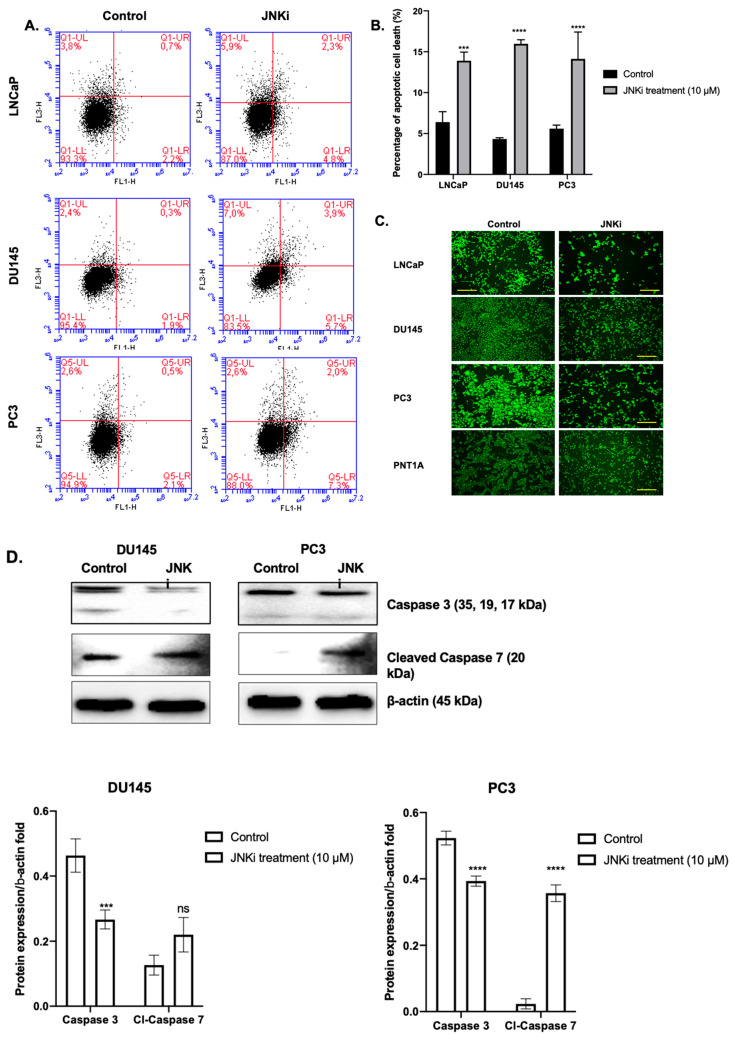
Inhibition of c-Jun N-terminal kinase (JNK) sensitizes LNCaP, DU145, and PC-3 cells to apoptosis. Flow cytometric analysis of 10,000 collected events for each experiment. (**A**) Evaluation by annexin-V staining and propidium iodide (PI) assay. Cells were treated for 48 h with JNK pathway inhibitor (JNKi). Then cells were stained with annexin-V–FITC/PI and examined by flow cytometry. (**B**) Abscissa: histogram of cells positive for annexin-V; ordinate: cells positive for PI uptake. Note the log scales—lower left quadrant shows viable cells; lower right, annexin-V positive cells (early apoptosis); upper right, cells positive for both annexin-V and PI (late apoptosis). (**C**) Inhibition of JNK reduces cell viability via induction of mitochondrial membrane permeabilization (MMP) loss in LNCaP, DU145, PC-3, and PNT1A cells. Cells were stained with DiOC6 (4 mM) following treatment of 48 h with JNKi. Then cells were visualized by fluorescence microscopy (Ex/Em: 488/ 525 nm; scale bar is 100 µm. (**D**) JNKi affects caspase-dependent cell death in DU145 and PC-3 cells. The Western blotting analysis was carried out with anti-caspase 3 and caspase 7; β-actin was used as a loading control.

Exposure of LNCaP, DU145, and PC-3PC-3 PCa cells to JNKi for 48 h slightly increased the apoptotic cell death ratio, which accompanied decreased mitochondrial activity in all PCa cell lines. JNK inhibition resulted in a 10% increase in cell death compared to control cells in PCa (Figure 1A and B, *n* = 3; *** *p* =0.00502 for LNCaP, **** *p* < 0.0001 for DU145 and PC-3 cells). Moreover, blocking the JNK pathway resulted in increased ROS generation in both DU145 and PC-3 PCa cells (Figure S1), which is an indicator of apoptosis. We then tested mitochondrial membrane permeabilization (MMP) changes and found that JNKi treatment did not lead to a significant alteration of PNT1A normal prostate epithelial cells, while it decreased MMP in all malignant cell lines by cell type-dependent manner changes in LNCaP, DU145, and PC-3 cells (Figure 1C). Thus, we concluded that JNKi treatment significantly reduced cell survival and viability of malignant PCa cells. We found that LNCaP cells were the more sensitive cell line within all PCa cells against JNKi treatment. We then wanted to confirm that JNKi induces apoptosis; indeed, it is noteworthy that JNKi treatment led to the activation of cleaved caspase 3/7 in DU145 and PC-3 PCa cells (Figure 1D, *n* = 3, DU145 cells: *** *p* = 0.0009 for caspase 3, *p* = 0.0539 for caspase 7; PC-3 cells, all conditions **** *p* < 0.0001 for DU145 and PC-3 cells).

**Figure 2:**
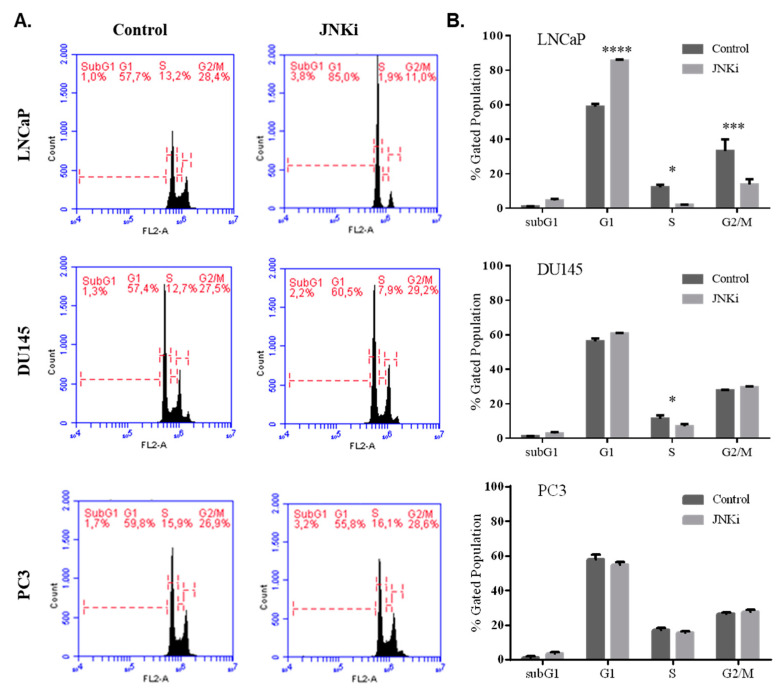
JNKi-induced cell cycle arrest was detected at the G1 phase in LNCaP, but not in DU145 and PC-3 cells. (**A**) DNA content was analysed by flow cytometry (right figures). (**B**) The percentage of cells in the G1, S, and G2/M phases of the cell cycle are shown in the graphics for each cell. The representative results are shown (* *p* = 0.03, ** *p* = 0.021, *** *p* = 0.0002, **** *p* < 0.0001; *n* = 3).

In accordance with these findings, JNKi treatment for 48 h increased cell cycle arrest at the G1 phase in LNCaP PCa cells, but not in DU145 and PC-3 cells (Figure 2A,B). Thus, we concluded that JNK inhibition for 48 h increased caspase activity to initiate cell death processes, while longer treatment is required to finalize cell death only for LNCaP cells, in contrast to more metastatic PC-3 PCa cells.

**Figure 3:**
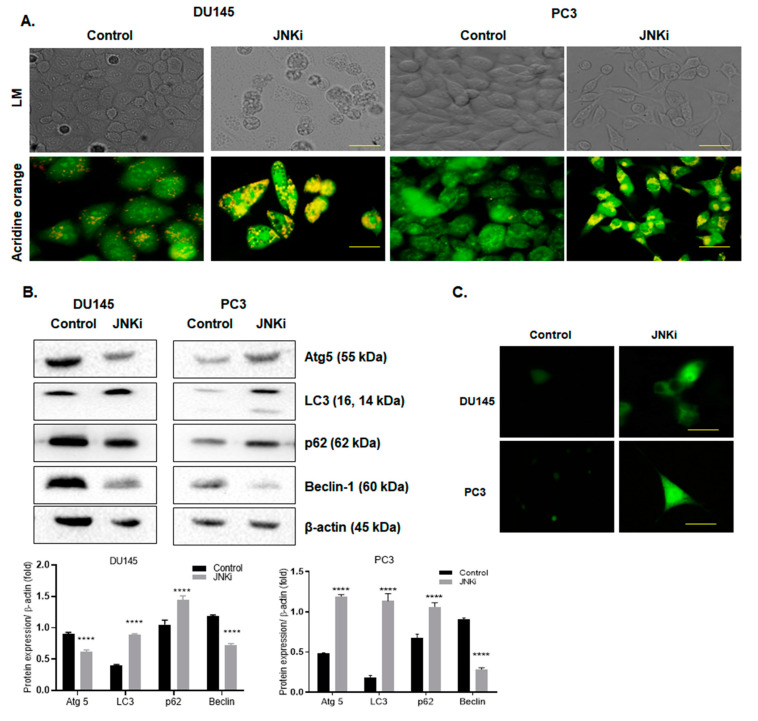
JNK inhibitor-induced autophagy via increasing vacuole formation. (**A**) Acridine orange staining was performed to visualize the acidic vacuole formations by fluorescence microscopy in DU145 and PC-3 cells. Scale bar is 10 µm. (**B**) The expression profile of autophagy markers, such as Atg5, LC3, p62, and Beclin-1 was determined by immunoblotting. β-actin was used as a loading control. Obtained-results were analyzed by Bonferroni’s multiple comparison test. **** *p* < 0.0001. (**C**) GFP–LC3 transfected cells were treated with JNKi for 48 h, and green dots were visualized under fluorescence microscopy. Scale bar is 10 µm.

Since we observed morphological changes in the cells following the JNKi, we wanted to test these changes further. Indeed, in this study, inhibition of the JNK pathway led to abnormal vacuole formation on both androgen-independent DU145 and PC-3 PCa cells (Figure 3A, *n* = 3). In a similar way, acidic vacuole formation, which was stained with acridine orange following JNKi treatment, was obvious in both cell lines (represented as green–yellow dots, Figure 3A). Similar results were obtained following lysotracker staining, which indicated that JNKi increased the acidic vacuoles in DU145 and PC-3 cells. However, we did not detect similar changes in vacuole formation of LNCaP cells (Figure S1). In order to better understand the autophagic effect of JNKi, due to increased vacuole formation, we then checked the expression level of key players of autophagy on AR-independent PCa cell lines. We determined that JNKi treatment enhanced LC3 lipidation in PC-3 cells. In accordance with this finding, JNKi-mediated accumulation of p62 and Atg5 upregulation was remarkable in its autophagic signatures, while JNKi treatment slightly increased LC3 and p62 expression levels in DU145 cells (Figure 3B; *n* = 3; *p* < 0.0001). This finding was also proved by GFP-tagged LC3 transfection, in which the expression of plasmid was promoted following JNKi treatment in both cell lines (Figure 3C).

**Figure 4:**
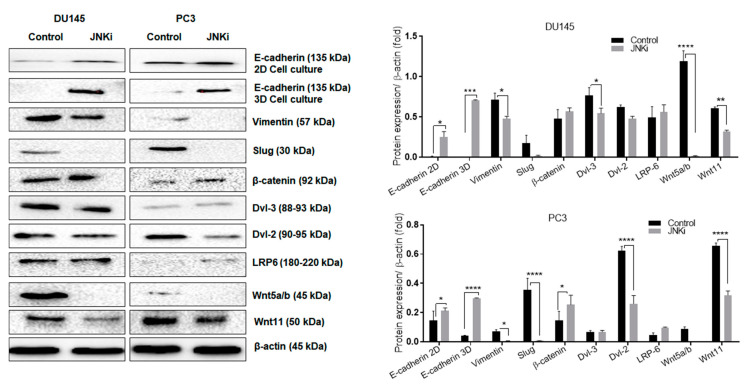
The inhibition of JNK reduces the epithelial–mesenchymal transition (EMT) process in both DU145 and PC-3 cells. Total protein isolation proceeded following JNKi treatment in DU145 and PC-3 cells. Additionally, the total protein was isolated from hanging drop spheroids in DU145 and PC-3 cells, as well as E-cadherin, vimentin, Slug, β-catenin, Dvl-3, Dvl-2, LRP6, and Wnt5a/b and Wnt11. β-actin was used as a loading control. Expression levels were analyzed by ImageJ and are presented as column graphics. DU145 E-cadherin (two-dimensional; 2D): * *p* = 0.0213; vimentin: * *p* = 0.0334; Dvl-3: * *p* = 0.0396, ** *p* = 0.0042, **** *p* < 0.0001; PC-3 E-cadherin (2D): * *p* = 0.022, Vimentin: * *p* = 0.0342, β-catenin: * *p* = 0.023, **** *p* < 0.0001.

In order to evaluate further the cellular characteristics of androgen independent cells in the presence of JNKi (Figure 4, we first checked E-cadherin expression levels in both two-dimensional (2D) and 3D cell culture growing environments. We detected that JNKi upregulated 2D and spheroid culture-mediated, 3D environment, PC-3 PCa cells (Figure 4). Spheroid formation findings are also presented in Figures S2 and S3. Long-term treatment of spheroids with JNKi was effective at reducing the diameter of spheroids. Thus, we conclude that the selected concentration of JNKi effectively reduced the aggressive phenotype of PCa cells. Moreover, as negatively correlated with E-cadherin protein expression, mesenchymal markers vimentin and Slug down-regulation was found after JNK inhibition. Long term treatment of spheroids with JNKi was effective at reducing the diameter of spheroids. Thus, we conclude that the selected concentration of JNKi effectively reduced the aggressive phenotype of PCa cells. In a similar way, β-catenin and LRP6 upregulation following JNKi in both PCa cells was detected. In accordance with these data, JNK inhibition reduced EMT via Wnt-11, Wnt5a/b, vimentin, and Slug down-regulation. Therefore, we concluded that JNK promoted EMT in DU145 and PC-3 cells. While JNK inhibitor treatment altered the dishevelled-3 (Dvl-3) and -2 expression levels in a cell type-dependent manner, the only downregulation for Dvl-2 was observed in PC-3 cells, whereas the Dvl-3 expression profile did not change (Figure 4). Similarly, following JNK inhibition we did not detect any significant change in lipoprotein receptor-related protein 6 (LRP6) on DU145 cells, whereas a significant slight increase was detected in PC-3 cells (*p* > 0.05; Figure 4).

### 3.2. Wnt-11 Promotes EMT in PCa Cells via JNK Signalling

**Figure 5:**
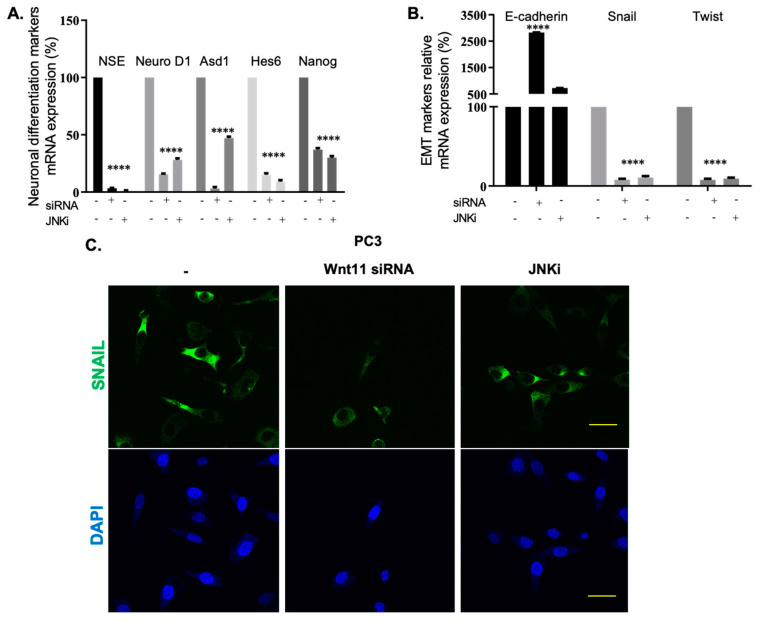
The effects of Wnt-11 on the neuronal differentiation and EMT markers in JNKi-treated PC-3 cells. PC-3 cells were transfected with Wnt-11 siRNA for 48 h. (**A**) The mRNA levels of NSE, Ascl1, Hes6, and Nanog in the control, as well as Wnt-11-silenced and JNKi-treated PC-3 cells were determined by RT-PCR (*p* < 0.0001). (**B**) The mRNA levels of E-cadherin, Snail, and Twist in PC-3 cells were determined by RT-PCR. Data from at least three independent experiments with duplicate treatments are shown (*p* < 0.0001). (**C**) Immunofluorescence localization of Snail in Wnt-11-silenced PC-3 cells and JNKi-treated-PC-3 cells compared to control cells. DAPI (4’,6-diamidino-2-phenylindole) was used for nuclear staining. Scale bar is 10 µm. Representative images of this experiment were taken from three biological repeats.

It has been suggested that JNK pathway mediates non-canonical Wnt-11 functions [35]. Since we established a link between cell death and JNK signalling, we then wanted to check whether the effect was regulated by Wnt-11. We have previously reported that Wnt-11 is involved in both NED and EMT differentiation in PCa [20,36]; however, how the signals are carried to the downstream target genes are unknown. To test whether the JNK pathway is responsible for Wnt-11 regulated cellular differentiation in PCa, we adopted PC-3 cells as a model, as they express the highest level of Wnt-11 mRNA. JNK inhibition resulted in the downregulation of NED markers, such as NSE, Ascl1, Hes6, and Nanog, and this effect was correlated with Wnt-11 siRNA in PC-3 cells (Figure 5A,B; *n* = 9, *p* < 0.01 for all; Figure S4: qRT-PCR data shows Wnt-11 mRNA expression decreased after silencing Wnt-11). The mRNA expression of mesenchymal markers like Twist and Snail was downregulated following either JNK inhibition or Wnt-11 silencing, and increased mRNA levels of the epithelial marker E-cadherin (Figure 5B; *n* = 9, *p* < 0.01 for all). We then tested this effect on the protein level and demonstrated that JNKi treatment significantly downregulated Slug, which is the transcriptional repressor of E-cadherin. Immunofluorescence staining was performed to determine the effect of both JNKi and Wnt-11 silencing on the localization and expression level of Snail in PC-3 cells. Correlated with mRNA results, Wnt-11 silencing, and inhibition of JNK significantly reduced the expression level of Snail in PC-3 cells (Figure 5C; *n* = 3). Therefore, we conclude that the JNK pathway involves cellular differentiation, and Wnt-11 is a positive regulator of mesenchymal markers via the JNK pathway.

### 3.3. Effect of Silencing Wnt-11 on Cellular Migration

**Figure 6:**
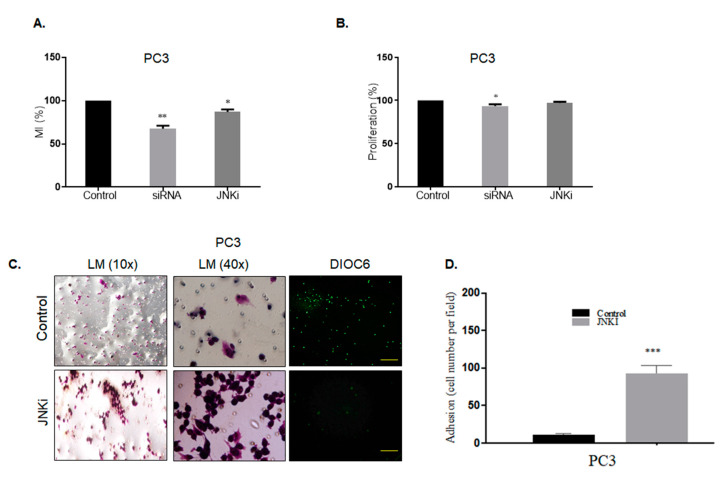
Effect of Wnt-11 silencing and JNKi on PCa cellular migration. (**A**) Motility index (MI) of PC-3 cells was evaluated by the transwell migration assay and presented as %. Migrated JNKi-treated PC-3 cells and Wnt-11-silenced PC-3 cells were counted (* *p* < 0.05; *n* = 3; ** *p* = 0.0026). (**B**) Proliferation assay was performed in JNKi-treated PC-3 cells and Wnt-11-silenced PC-3 cells (*n* = 3; * *p* = 0.0486). (**C**) A Boyden chamber assay was performed following JNKi treatment in PC-3 cells. Unmigrated cells were visualized by crystal violet staining, and (**D**) cells were counted by fields that were randomly selected at ×10 magnification. Migrated cells were stained with DiOC6 and observed with fluorescence microscopy. The scale bar is 100 µm; *** *p* = 0.002.

Previously, we had reported that the downregulation of Wnt-11 reduced cell migration and invasion in PCa [36]. We wanted to clarify whether this effect of Wnt-11 was JNK pathway-dependent. For this reason, the most metastatic PC-3 cells were used as a model, and cell motility and the invasion of PC-3 cells was studied by using transwell migration and Boyden chamber invasion assays. Silencing Wnt-11 resulted in significant suppression of transwell cell migration after 6 h by 35 ± 3%, as expected, and JNK inhibition reduced cell motility by 15 ± 4% (*n* = 5, *p* < 0.01 for both; Figure 6A). In contrast, both of the treatments showed that there was no effect on proliferative activity of cells following 6 h treatment (Figure 6B). Moreover, JNK inhibition decreased the invasion of PC-3 cells significantly when compared with untreated cells (*n* = 5, *p* = 0.002; Figure 6C–D).

**Figure 7:**
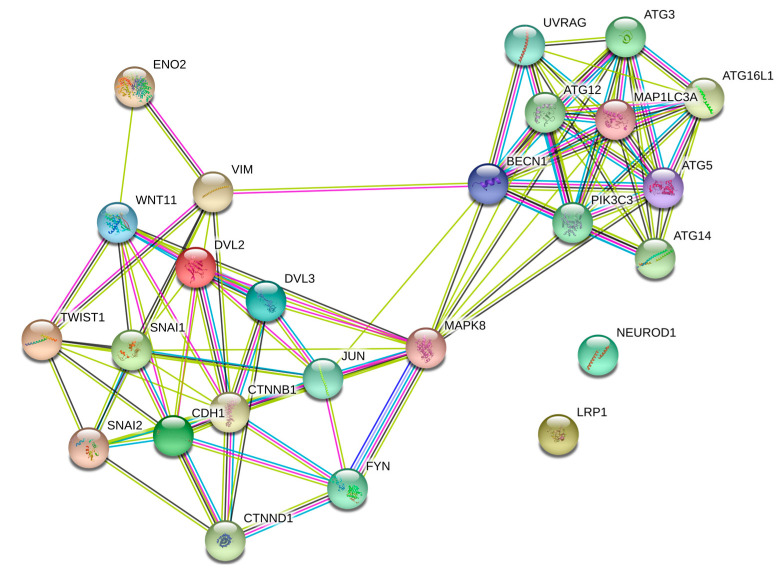
The putative binding partners of Wnt-11 and JNK were shown by using STRING (Search Tool for the Retrieval of Interacting Genes/Proteins) analysis (https://string-db.org/; STRING combined score > 0.4) [37]. Lines between nodes represent known interactions (from curated databases: blue; experimentally determined: pink), predicted interactions (gene neighbourhood: green; gene fusions: red; gene co-occurrence: blue) and other interactions (text mining: lime green; co-expression: black; protein homology: grey). Red and green circles represent query proteins and the first shell of interactors’ functional involvement of proteins.

Our findings indicate that JNK inhibition limits the EMT in PCa cells through a similar pathway as Wnt-11. STRING (Search Tool for the Retrieval of Interacting Genes/Proteins, https://string-db.org/ [37]) analysis for Wnt-11 and JNK is shown in Figure 7; JNK is a strong mediator between Wnt-11-driven EMT progression and autophagy responses in the cells.

## 4. Discussion

The main results of the present study are as follows: (1) PCa cell lines showed different responses against JNKi treatment; (2) Wnt-11 promoted NED expression and EMT, cell motility, and migration without affecting proliferative activity via the JNK pathway.

### 4.1. JNKi Modulates the Survival and Death Axis in a Cell-Type Dependent Manner

JNK pathway activation has been shown to have pivotal roles during drug-induced cell death [38,39]. It is known that JNK activity has regulatory roles in ROS-induced cellular responses, and the downregulation of JNK activation has also been shown to lead to inhibition of apoptosis in PCa cells by androgens [40]. Moreover, the FDA approval of JNK inhibitors in the treatment of melanoma opens up a new era for their therapeutic potential for other cancers, which exert active JNK signalling [41]. In the current study, inhibition of JNK induced PCa cell death through MMP loss and an increase in caspase activation in a cell type-dependent manner. Although JNKi did not alter cell viability and mitochondrial activity in PNT1A prostate epithelial cells, LNCaP PCa cells were found in the sensitive cell line compared to DU145 and PC-3 cells. This is in line with findings from a previous study showing that treatment with JNK inhibitors in A549 lung cancer cells also induced MMP loss and increased intracellular ROS levels through the depletion of glutathione [42]. Thus, the inhibition of JNK is a powerful strategy to disrupt cell viability and survival functions in the cancer cells. However, we found that JNK inhibition only induced the cell cycle arrest at the G1 phase in LNCaP cells, but not in DU145 and PC-3 prostate cancer cells. These cells were found with autophagic vacuoles following JNK inhibition. In the previous studies, it was shown that JNK-mediated autophagy is controversial, according to different stress indicators. Increased ROS generation was shown to be an important mediator in cellular autophagy formation, which may resist apoptosis in mesenchymal stem cells through Beclin-1. Beclin-1 could bind to anti-apoptotic Bcl-2 with the presence of JNK. However, elimination of JNK signalling in the cells may cause the rapid induction of autophagic responses to render drug-mediated cell death responses in the cells [43]. On the contrary, it has been shown that JNK inhibitor-mediated autophagy may increase the apoptotic effect of mTOR inhibitors in non-small-cell lung cancer cells [44].

### 4.2. JNK and Wnt-11 Presented Similar Functional Properties in the Control of Metastasis-Associated Biomarkers

Recent studies have focused on the potential relationship between autophagy and EMT-related signalling pathways, in order to clarify the role of autophagy on metastasis and differentiation [45]. TGFβ promotes pro-autophagy gene expression and results in the activation of convergent signalling targets like p38 and JNK. Similar to this effect, TGFβ enhances the Wnt-11 signalling and activates JNK in the cells to main cellular differentiation [46]. According to our previous studies, the increased Wnt-11 mRNA expression correlates with metastatic potential and hormone dependency in PCa cell lines and xenograft models [18,20]. Here, we tested the effect of JNK signalling on PCa metastasis by using JNKi on PC-3 cells that express the highest level of Wnt-11. JNK inhibition prevents E-cadherin loss in both 2D and 3D cell models, which is promising in the treatment of metastatic phenotype of cancer cells. Concomitant downregulation of Vimentin and Slug following JNKi treatment showed that JNK-mediated EMT could be prevented by the treatment of JNK inhibitors. We determined that JNK inhibition increased β -catenin expression levels in both DU145 and PC-3 cells. It has been shown that JNK mediates β-catenin phosphorylation and E-cadherin/β-catenin disrupts cell contacts, and adherence could be prevented by the treatment of JNK siRNA [47].

Interestingly, in our study, we found that JNKi diminished the expression level of another non-canonical Wnt member, Wnt5a/b, both in DU145 and PC-3 cells. Previously, it was reported that Wnt5a overexpression enhanced the invasion of PC-3 cells [48]. Another study highlighted the importance of Wnt5a expression following AR inhibition by using single-cell RNA sequencing of circulating tumour cells of metastatic PCa patients, and concluded that a subpopulation of PCa cells expresses non-canonical Wnts to enhance their survival [49]. Moreover, all Wnt proteins are shown to interact with the same receptors and co-receptors, such as LRP5/6 [50]. In this study, we found that the LRP6 protein level was not altered after blocking the JNK pathway. Wnt and its coreceptors LRP5/6 and Frizzled (Fz) at the plasma membrane initiated the canonical signalling axis to translocate β-catenin into the nucleus via degradation of the Axin complex. JNK inhibition concomitantly increased β-catenin accumulation in the cells. Moreover, we have found that the suppression of JNK enhances intracellular protein Dvl-2 in PC-3 cells, but not in DU145 cells. This is interesting, as Dvl plays a dual role in both canonical and non-canonical pathways, and has been termed as a molecular switch between them [51]. Moreover, it has been suggested that JNK lies downstream of Dvl in flies while Wnt-11 mediated activation of noncanonical Wnt signalling through PKC and JNK is required for heart development in Xenopus [52]. Dvl2 was also reported as a PCa-associated gene, and it has been suggested that its expression may increase PCa metastatic potential [53]. Our data suggest that non-canonical Wnt pathway members Wnt-11 and Wnt5a/b are more expressed in AR-independent, more metastatic PC-3 cells, and JNKi reduces their metastatic potential via Dvl2.

Moreover, we have demonstrated that Wnt-11 controls differentiation and cell motility signals via the JNK pathway. Wnt family members are known to play regulatory roles in metastasis-related physiological characteristics, such as stemness, EMT, cell differentiation, and invasiveness in malignancies; Wnt-11 mRNA expression correlates strongly with levels of NED in cancers of the prostate and breast [7,19,20,54]. Moreover, it has been reported that an active JNK status of cells has an impact on cell survival under the effects of androgens in PCa progression [40]. Thus, either silencing Wnt-11 or treating cells with the JNK blocker decreased the expression level of several biomarkers that are involved in metastasis, in particular NEDs or those involved in EMT both in mRNA and protein levels. The inhibition of JNK led to the upregulation of E-cadherin and the downregulation of Vimentin and Slug in AR-independent PCa cells PC-3 and DU145. These results were similar to previous studies, which showed that inhibition of JNK decreased EMT in HCT116 colon carcinoma cells [55]. JNK inhibition was shown to decrease hypoxia-mediated EMT transition and stemness properties in HT29 and SW-480 colon carcinoma cells [56].

Interestingly, the inhibitory effect of the JNK pathway on cell migration was seen without any change in proliferative activity in short-term treatment for 6h. The sphere-formation capacity of cancer cells is a widely accepted marker for the estimation of the stemness properties of cells. Similar to previous findings, we have shown that JNKi recurrent administration reduces the diameter of spheroids in long-term treatment modalities in DU145 and PC-3 cells [57]. This is consistent with the notion that primary and secondary tumorigenesis (i.e., proliferation vs. migration) can be controlled differently, even independently [54]. These findings constitute strong evidence that Wnt-11 promotes the migratory behaviour of cells, including multipotent stem/progenitor cells, during development and cancer progression, including in cancers of breast, prostate, colon, and cervix [19,20,21,54]. Wnt-11 is regulated by TGFβ and calcium in a wide variety of cell types, and its activation is triggered by β-catenin-dependent or independent pathways, highlighting the potential role of β-catenin in JNK-mediated EMT and neuroendocrine signalling axes. Similar findings were also shown in invertebrates, which indicates that Wnt-11 has a promising role under control of the Wnt/β-catenin signalling axis in the differentiation of cells during the early development of the eye in Zebrafish and *Drosophila* models [58,59,60].

## 5. Conclusions

This study shows that JNK promotes autophagy and EMT in androgen-independent PCa cells. Extended JNKi treatment prevented colony formation and an increase in apoptotic cell death in DU145 and PC-3 cells via alteration of MMP and ROS generation. The role of Wnt-11 in the pathophysiology of PCa was furthermore shown to be mediated via JNK signalling. Therefore, further research should be focused on this interaction, in order to evaluate the central roles of both the JNK pathway and Wnt-11 to understand PCa prognosis. Signals produced from these pathways may initiate the phenomenon of EMT and NED, and thus, novel combinatorial therapies effectively targeting JNK and Wnt-11, alongside other signalling mechanisms, may provide more effective treatment for PCa.

## Supplementary Materials

The following are available online at https://www.mdpi.com/2079-7737/9/7/142/s1, Figure S1. JNK inhibition (10 µM) resulted in an increased ROS generation and acidic vacuole formation in both DU145 and PC-3 PCa cells. Figure S2. JNK inhibitor decreased 3D spheroid growth and invasive potential of DU145 cells within 96 h. Figure S3. JNK inhibitor (10 µM) decreased anchorage-independent growth of DU145 and PC3 prostate cancer cells. Figure S4. Real-time qPCR result of Wnt-11 relative mRNA expression in PC3 cells.
