# Supplementary material for: Inhibition on JNK Mimics Silencing of Wnt-11 Mediated Cellular Response in Androgen-Independent Prostate Cancer Cells
Source: Biology (Basel). 2020 Jun 27;9(7):142. doi: 10.3390/biology9070142 (PMC7407974; doi:10.3390/biology9070142)
Supplement: Supplementary file 1 [file biology-09-00142-s001.pdf]

Supplemental Figure 1

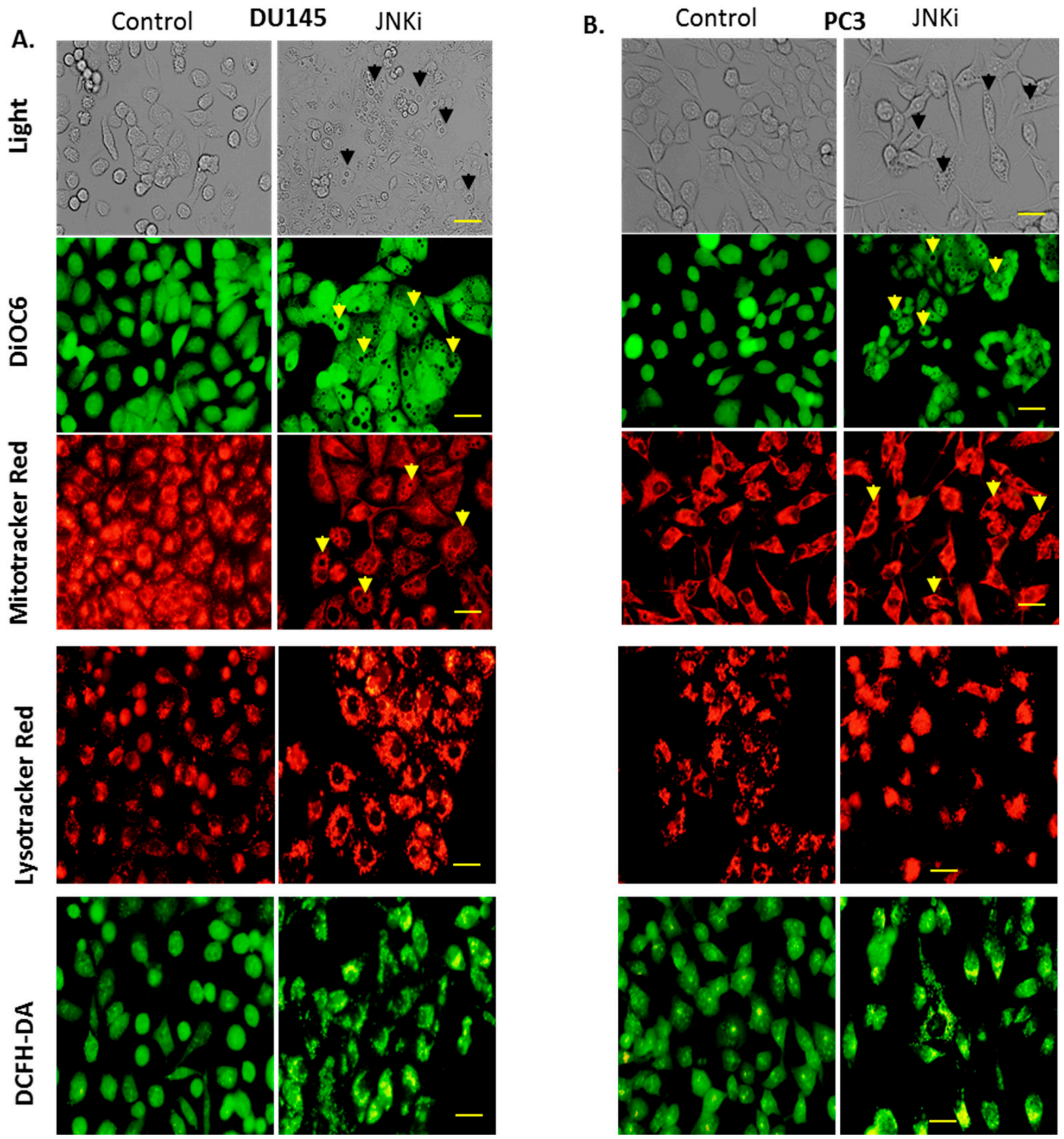

Figure S1. JNK inhibition (10 μM) resulted in an increased ROS generation and acidic vacuole formation in both DU145 and PC-3 PCa cells. Thel vacuole formation following JNKi treatment was examined in DU145 (A) and PC3 (B) through light and fluorescence microscopy (40 ×) after DiOC6 and MitoTracker red staining. For MitoTracker Red staining: Ex/Em = 561/620 nm. Scale bar: 10μm. Lysotracker red (Ex/Em = 577/590 nm) staining was performed to visualize the acidic vacuoles following JNKi treatment. ROS generation was determined by DCFH-DA (Ex/Em = 485/535 nm) staining in each cell line. Representative images of this experiment were taken from three biological repeats.

Supplemental Figure 2

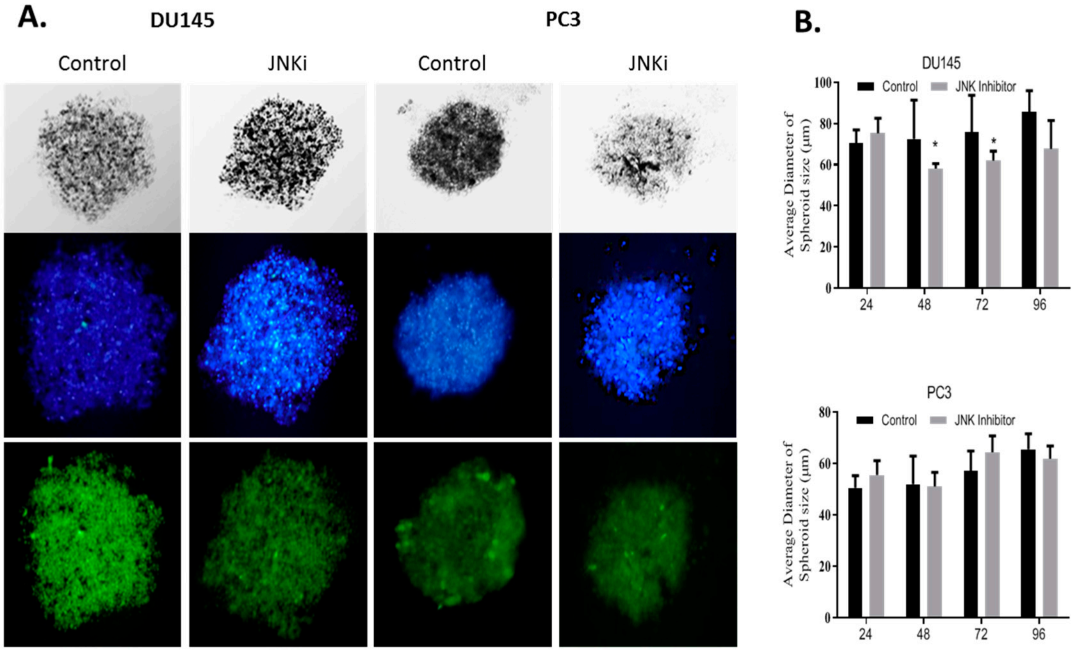

Figure S2. JNK inhibitor decreased 3D spheroid growth and invasive potential of DU145 cells within 96 h. Both cell lines were seeded at a density $25x10^2$ through hanging drop method in the presence or absence of JNK inhibitor. (A) DiOC6 and DAPI co-staining was performed to evaluate the live and dead cells within colony following 96 h treatment. (B) Every 24 h diameter of each colony was calculated via microscopy images. Column graphs represents the Mean±Std.Dev of ten different colonies, n=3.

Supplemental Figure 3

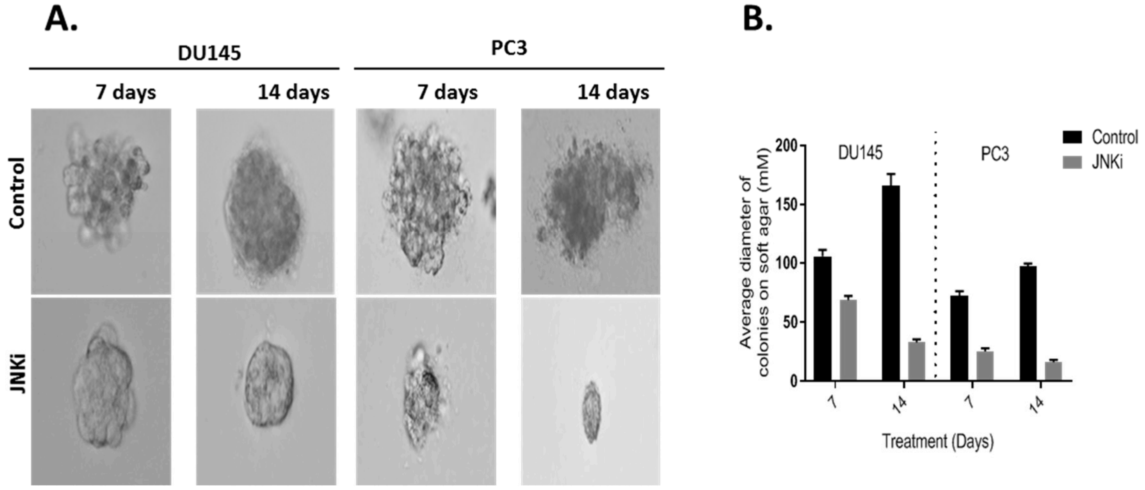

Figure S3. JNK inhibitor (10 µM) decreased anchorage independent growth of DU145 and PC3 prostate cancer cells within 14 days. (A) Both cell lines were seeded at a density $1x10^4$ through soft agar method in the presence or absence of JNK inhibitor. (B) Every 7 days diameter of each colony was calculated via microscopy images. Column graphs represents the Mean±Std.Dev of eight different colonies, n=3.

Supplemental Figure 4

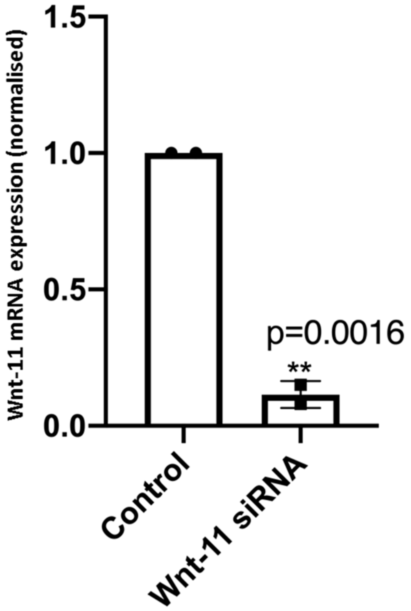

Figure S4. Real-time qPCR result of Wnt-11 relative mRNA expression in PC-3 cells. Data are plotted as fold-differences relative to the mock/control siRNA of transfected PC-3. Wnt-11 mRNA expression level after normalising with housekeeping gene (RPII) level, Wnt-11 siRNA reduces the expression 12.5 fold ($n=6$; $p=0.0016$). All data were analysed as means ± standard errors.
